# The Mechanism of the Anti-Obesity Effects of a Standardized *Brassica juncea* Extract in 3T3-L1 Preadipocytes and High-Fat Diet-Induced Obese C57BL/6J Mice

**DOI:** 10.3390/nu16060846

**Published:** 2024-03-15

**Authors:** June-Seok Lim, Ji-Hyun Im, Xionggao Han, Xiao Men, Geon Oh, Xiaolu Fu, Woonsang Hwang, Sun-Il Choi, Ok-Hwan Lee

**Affiliations:** 1 Department of Food Science and Biotechnology, Kangwon National University, Chuncheon 24341, Republic of Korea; dlawnstjr725@naver.com; 2 Department of Food Biotechnology and Environmental Science, Kangwon National University, Chuncheon 24341, Republic of Korea; ijh108020@gmail.com (J.-H.I.); xionggao414@hotmail.com (X.H.); menxiaodonglei@naver.com (X.M.); dhrjs1@gmail.com (G.O.); fuxiaolu2019@gmail.com (X.F.); 3 STR Biotech Co., Ltd., Chuncheon 24232, Republic of Korea; hwang931@strbiotech.co.kr

**Keywords:** standardized *Brassica juncea*, 3T3-L1, C57BL/6J, anti-obesity, mechanism

## Abstract

Obesity is a global health concern. Recent research has suggested that the development of anti-obesity ingredients and functional foods should focus on natural products without side effects. We examined the effectiveness and underlying mechanisms of *Brassica juncea* extract (BJE) in combating obesity via experiments conducted in both in vitro and in vivo obesity models. In in vitro experiments conducted in a controlled environment, the application of BJE demonstrated the ability to suppress the accumulation of lipids induced by MDI in 3T3-L1 adipocytes. Additionally, it downregulated adipogenic-related proteins peroxisome proliferator-activated receptor-γ (PPAR-γ), CCAAT/enhancer-binding protein-α (C/EBP-α), adipocyte protein 2 (aP2), and lipid synthesis-related protein acetyl-CoA carboxylase (ACC). It also upregulated the heat generation protein peroxisome proliferator-activated receptor gamma coactivator-1α (PGC-1α) and fatty acid oxidation protein carnitine palmitoyltransferase-1 (CPT-1). The oral administration of BJE decreased body weight, alleviated liver damage, and inhibited the accumulation of lipids in mice with diet-induced obesity resulting from a high-fat diet. The inhibition of lipid accumulation by BJE in vivo was associated with a decreased expression of adipogenic and lipid synthesis proteins and an increased expression of heat generation and fatty acid oxidation proteins. BJE administration improved obesity by decreasing adipogenesis and activating heat generation and fatty acid oxidation in 3T3-L1 cells and in HFD-induced obese C57BL/6J mice. These results suggest that BJE shows potential as a natural method for preventing metabolic diseases associated with obesity.

## 1. Introduction

Obesity is characterized by the abnormal and excessive buildup of fat in adipose tissue, leading to an unhealthy condition, as outlined by the World Health Organization (WHO) [1,2]. The body mass index (BMI) of an individual is calculated by dividing their weight in kilograms by the square of their height in meters. A BMI score that falls within the range of 25 to 30 is categorized as being overweight or obese [1,2,3]. Based on the findings of the WHO’s report on obesity, it has been indicated that there are approximately 1.9 billion adults over the age of 18 who are classified as overweight, with an additional 600 million falling into the category of obesity. These data underscore the significant global prevalence of obesity as a pressing public health concern [4,5]. Based on data from the National Health and Nutrition Examination Survey conducted in Korea, it was found that the incidence of obesity in individuals aged 19 or older was reported as an estimate of approximately 26% for women and 40% for men. By 2030, its prevalence is anticipated to reach approximately 37% in women and 62% in men [6].

The primary cause of obesity is excessive accumulation of body fat resulting from caloric intake over the body’s energy consumption [7]. Adipocytes function as endocrine organs by regulating several metabolic processes that contribute to physiological regulation. These cells release a range of hormones, including leptin and adiponectin, which play essential roles in the maintenance of metabolic homeostasis [8]. The excessive buildup of lipids within adipocytes results in impaired functionality, including issues like mitochondrial dysfunction and intracellular endoplasmic reticulum stress. This can lead to insulin resistance, inflammatory reactions, and an increased susceptibility to complications associated with obesity, such as cardiovascular disease, hypertension, fatty liver, and cancer [9,10].

Lipid accumulation occurs due to increased adipocyte differentiation and triglyceride production during adipogenesis [11]. The accumulation of lipids regulates the dimensions of lipid droplets via the augmentation of cell count or proliferation [12]. The 3T3-L1 cell line, isolated from a mouse embryo, is frequently used in obesity-related in vitro studies [13]. This cell line can differentiate into adipocytes when treated with 3-isobutyl-1-methylxanthine (IBMX), dexamethasone (Dex), and insulin, which stimulate complex signaling pathways and various transcription factors [14,15]. The various transcription factors include CCAAT/enhancer-binding protein α (C/EBP-α), nuclear receptor peroxisome proliferator-activated receptor-γ (PPAR-γ), and adipocyte fatty acid-binding protein (aP2) [16]. In the advanced phase of cellular differentiation, there is a rise in the concentrations of C/EBP-α in conjunction with PPAR-γ, resulting in gene stimulation that promotes adipogenesis, such as aP2 [17,18]. These factors show significantly increased expression levels in differentiated adipocytes [19]. Controlling the expression of factors linked to the differentiation of adipocytes is an important approach for preventing and treating obesity [20]. Therefore, therapeutic agents demonstrating efficacy in promoting adipocyte differentiation and inhibiting adipogenesis have been developed. Currently, the drugs targeting obesity include orlistat, topiramate, sibutramine, rimonabant, and phenylpropanolamine. However, these drugs come with adverse reactions such as sleeplessness, loss of appetite, digestive issues, elevated blood pressure, and heart disease [21]. To address this concern, active research is dedicated to creating treatments for obesity by utilizing safer natural substances or phytochemicals that have minimal adverse reactions [22].

Leaf mustard (*Brassica juncea*) is a cruciferous vegatable that is commonly grown in Korea and Japan and has a history of being utilized in kimchi as an ingredient in Korea [23]. The nutritional components of *Brassica juncea* are rich in various free sugars, fatty acids, amino acids, vitamins, and minerals, and it has been documented to exhibit exceptional antioxidant activity in cell-based studies [24]. Moreover, glucosinolates and isothiocyanate compounds found in Brassica juncea, such as allyl isothiocyanate and sinigrin, are recognized for their diverse health benefits and functional properties, including their potential anti-cancer effects [25,26,27]. Sinigrin has been reported to demonstrate anticancer, anti-inflammatory, antibacterial, antifungal, and antioxidant effects. Our previous study reported that *Brassica juncea* extract (BJE), extracted under optimized sinigrin extraction conditions, has anti-obesity effects in vitro [28,29,30]. Furthermore, there have been reports indicating that BJE exhibits anti-obesity properties in living organisms; however, research on the specific underlying mechanism remains limited [31]. Based on previous reports, we hypothesized that BJE induces anti-obesity effects via the regulation of adipogenesis, lipid synthesis, heat generation, and fatty acid oxidation. In accordance with our hypothesis, we investigated the mechanism of how BJE exerts its anti-obesity effect in vivo and demonstrated its value as a natural product with anti-obesity efficacy.

## 2. Materials and Methods

### 2.1. Chemicals and Standards

Bovine serum (BS), penicillin–streptomycin (P/S), trypsin–ethylenediaminetetraacetic acid (trypsin-EDTA), Dulbecco’s modified Eagle’s medium (DMEM) with high glucose, phosphate-buffered saline (PBS), and fetal bovine serum (FBS) were purchased from Gibco (Gaithersburg, MD, USA). The primary and secondary antibodies specific for peroxisome proliferator-activated receptor gamma coactivator-1α (PGC-1α), PPAR-γ, acetyl-CoA carboxylase (ACC), C/EBP-α, phospho-ACC (p-ACC), aP2, and carnitine palmitoyltransferase-1 (CPT-1) were purchased from Santa Cruz Biotechnology, Inc. (Dallas, TX, USA), and Cell Signaling Technology, Inc. (Danvers, MA, USA). Insulin, Dex, IBMX, and sinigrin were purchased from Sigma-Aldrich Co. (St. Louis, MO, USA).

### 2.2. Standardized BJE Preparation

*Brassica juncea* was provided by STR Biotech Co., Ltd. (Chuncheon, Republic of Korea). In prior research, we established a uniform extraction technique by conducting methodical experiments that focused on variables such as extraction duration, type of solvent used, and extraction temperature. Briefly, we adopted a standardized experimental method using 60% ethanol at 70 °C as the extraction solvent, and the extraction time was 3 h [30]. The materials utilized in the experiment were subjected to a washing procedure to eliminate any unwanted components, followed by freeze-drying using a freeze dryer (Ilshin, Seoul, Republic of Korea). The samples were subsequently pulverized and homogenized in preparation for their utilization in the experiment. Ethanol (60%) was added to 40 kg freeze-dried *Brassica juncea*. After subjecting *Brassica juncea* to reflux extraction at a temperature of 70 °C for a duration of 3 h, the resulting extraction solution underwent filtration using a 0.2 μm filter paper. The extract was evaporated under reduced pressure at 50 °C using a vacuum evaporator and then freeze-dried to produce BJE powder. The prepared sample was stored at a temperature of −20 °C and subsequently utilized following suspension in DMSO.

The concentration of sinigrin in BJE was quantified via High-Performance Liquid Chromatography (HPLC). The instruments used for analysis were a Shimadzu LC system (LC-40B XR, Shimadzu Co., Ltd., Kyoto, Japan) and a photodiode array detector (SPD-M40, Shimadzu Co., Ltd., Kyoto, Japan). Table 1 lists the conditions used in this analysis. The column used for analysis was CAPCELL PAK C_18_ UG120 (4.6 × 250 mm, 5.0 μm; Waters Corporation, Milford, MA, USA).

### 2.3. Cell Culture and XTT Assay

3T3-L1 pre-adipocyte cells (CL-173, ATCC, Manassas, VA, USA) were seeded at a concentration of 2 × 10^6^ cells/mL per plate according to the purpose of the experiment. The cells were then incubated in DMEM supplemented with 1% P/S and 10% BS until reaching full confluence. After 2 d of reaching full confluence, adipocyte differentiation was induced by a medium containing 1% P/S, 10% FBS, and a differentiation-inducing cocktail consisting of 0.5 mM IBMX, 1 μM Dex, and 1 μg/mL insulin. The medium for differentiation was replaced every 2 d containing 1 μg/mL insulin. BJE and Garcinia cambogia extracts (Gar) were added to the cells 2 d after reaching 100% confluence and treated continuously during differentiation. The total differentiation period was 6 d. 3T3-L1 adipocytes were cultured in a 96-well plate, and after 6 d of differentiation, a solution combining 1 mL of 2,3-bis-(2-methoxy-4-nitro-5-sulfophenyl)-2H-tetrazolium-5-carboxanilide (XTT) reagent with 20 μL of phenazine methosulfate reagent was prepared. This solution was then added to each well at a concentration of 20% of the medium volume.

### 2.4. Oil Red O Staining

Following a 6 d differentiation period, the culture medium was removed. 3T3-L1 cells that had undergone differentiation were rinsed two times with PBS and then fixed at room temperature for 1 h using 500 μL of a 10% formalin. After removing 10% formalin, the cells underwent a rinsing process with 60% isopropanol and subsequent air-drying at room temperature. After drying, the lipids accumulated in the cells were subjected to staining using a pre-prepared Oil Red O (ORO) working solution, with a ratio of ORO to DW of 6:4, for a duration of 1 h. The cells were rinsed thrice with DW and subsequently dried. ORO bound to lipids was separated by elution with 100% isopropanol, subsequently transferred to a 96-well plate, and absorbance was measured at 490 nm utilizing a microplate reader.

### 2.5. Animal Experiment

Male C57BL/6J mice, aged four weeks, were procured from DBL Inc. (Incheon, Republic of Korea), allowed to adjust to a controlled environment with specific conditions (temperature, 24 ± 5 °C; relative humidity, 55 ± 5%), and provided with ad libitum access to water and food within a 12/12 h light/dark cycle for a period of 1 week. Via a random selection process, mice were allocated into five groups: group 1, the control group that consumed 10% kcal normal-fat diet (CON); group 2, 60% kcal high-fat diet (HFD; D12492; Research Diet, Inc., New Brunswick, NJ, USA); group 3, HFD + Gar 50 mg/kg/day; group 4, HFD + BJE 400 mg/kg/day; and group 5, HFD + BJE 800 mg/kg/day. BJE and Gar dissolved in DW were administered orally once a day for 6 weeks. Food intake was evaluated by determining the difference between the amount of food given and the amount left over, and the food efficiency ratio (FER) was determined by dividing the weight gain (measured in grams) by the amount of food intake (measured in grams). Following a 12 h fasting period, mice were anesthetized with isoflurane (Hwaseong, Republic of Korea) at the conclusion of the breeding period. Following this, samples of tissue and blood were gathered for examination. Subsequently, samples of tissue and blood were collected for analysis. Blood samples were obtained from the orbital venous plexus of each mouse. The epididymal adipose tissue (eWAT), liver, spleen, and kidneys were dissected and rinsed with normal saline, and their weights were recorded. This study was approved by the Institutional Animal Care and Use Committee of the Kangwon National University (approval number: KW-221104-2).

### 2.6. Biochemical Analysis

Blood samples were collected in serum separation tubes (BD Microtainer^®^ tubes, 365967, BD Biosciences, Franklin Lakes, NJ, USA) and left to coagulate at room temperature for a duration of 30 min. The serum was isolated via centrifugation at a speed of 12,000× *g* and a temperature of 4 °C for a duration of 10 min. The levels of alanine aminotransferase (ALT), aspartate aminotransferase (AST), total cholesterol (TC), and total triglyceride (TG) in serum were assessed utilizing an automated biochemical analyzer (Hitachi-720, Hitachi Medical, Tokyo, Japan).

### 2.7. Histological Analysis

The eWAT was partially excised and fixed in 10% paraformaldehyde. The fixed eWAT was sequentially dehydrated with a graded series of ethanol solutions using a tissue processor (TP1020; Leica Biosystems, Nussloch, Germany) and paraffin (Rotary Microtome RM2255; Leica Biosystems). Paraffin blocks were cut to a thickness of 6 μm; slides were prepared and stained with hematoxylin and eosin (H&E). The stained adipose tissue was observed using an optical microscope (ECLIPSE Ni-U; Nikon, Melville, NY, USA).

### 2.8. Western Blot Analysis

eWAT were dissolved in lysis buffer containing 0.1% SDS, 150 mM sodium chloride, 1 mM pepstatin, 50 mM Tris-HCl, 1% Nonidet P-40, 0.25% sodium deoxycholate, and 1 mM phenylmethanesulfonyl fluoride, and reacted at 4 °C for 30 min. The cell lysates underwent centrifugation at 12,000× *g* and 4 °C for 20 min. The protein concentrations of the resultant cell lysates were quantified utilizing the Bradford protein assay kit (Bio-Rad Laboratories, Inc., Hercules, CA, USA). Equivalent amounts of proteins were separated using a 10% sodium dodecyl sulfate–polyacrylamide gel (SDS–PAGE) and electrotransferred onto a polyvinylidene difluoride membrane (0.2 μM Immun-Blot PVDF membrane; Bio-Rad Laboratories, Inc., Hercules, CA, USA). Tris-buffered saline containing 5% bovine serum albumin was used to inhibit nonspecific binding. The target proteins, after treatment with primary and secondary antibodies, were detected using Supersignal West Pico chemiluminescence (Thermo Fisher Scientific, Inc., Waltham, MA, USA). The Western blot bands were visualized with the ChemiDoc image software version 5.2.1 (Bio-Rad Laboratories, Inc., Hercules, CA, USA).

### 2.9. Statistical Analysis

The results are expressed as mean ± standard deviation (SD). All statistical analyses were performed using SPSS software (version 24.0; SPSS Inc., Chicago, IL, USA). Body weight, food efficiency ratio (FER), organ weights, and biochemical parameters were verified using Dunnett’s *t*-test for statistical significance, and cell viability, lipid accumulation, and Western blot results were verified using Duncan’s multiple range test for statistical significance. *p*-values less than 0.05 were considered statically significant.

## 3. Results

### 3.1. Sinigrin Content in Standardized BJE

**Figure 1:**
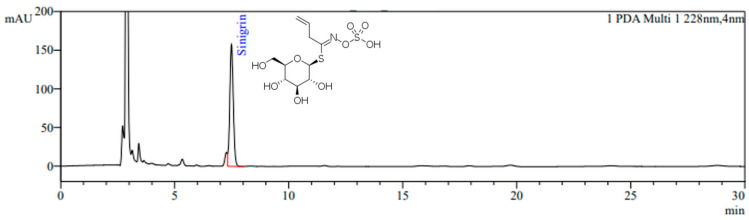
HPLC chromatogram of BJE. The monitoring wavelength for sinigrin was set at 228 nm.

The HPLC chromatogram of BJE is shown in Figure 1. Sinigrin was detected at a retention time of 7.4 min, and the sinigrin content in BJE was quantified as 18.0 mg/g.

### 3.2. Effects of BJE on the Viability and Lipid Accumulation of 3T3-L1 Adipocytes

**Figure 2:**
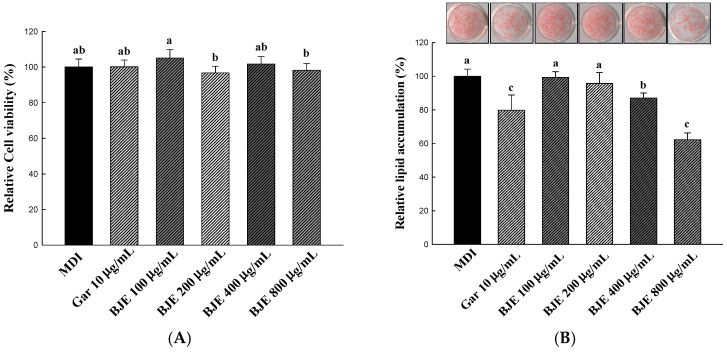
Effect of BJE on cell viability (**A**) and lipid accumulation (**B**) on different 3T3-L1 adipocytes. All results are presented as the mean ± SD of three independent in triplicate. Bars with different letters indicate significant differences at *p* < 0.05 using Duncan’s multiple range test.

The effect of BJE on cell viability during the induction of 3T3-L1 preadipocyte differentiation is shown in Figure 2A. The cells in the BJE treatment group (100–800 μg/mL) did not exhibit a statistically significant variance in comparison to the MDI group, indicating the absence of cytotoxic effects. Hence, this research aimed to assess the anti-obesity properties linked to the suppression of adipocyte differentiation by administering BJE at concentrations varying from 100 to 800 μg/mL. The impact of BJE on lipid accumulation in adipocytes was assessed via the application of Oil Red O staining. The effects of BJE treatment on lipid accumulation were examined by promoting the differentiation of 3T3-L1 cells over a 6 d duration (Figure 2B). A significant decrease in lipid accumulation was noted solely at BJE concentrations of 400 and 800 μg/mL, while no notable impact was detected at concentrations of 100 or 200 μg/mL. Additionally, lipid accumulation significantly decreased as the BJE concentration increased from 400 to 800 μg/mL.

### 3.3. Effect of BJE on the Regulation of Protein Expression Associated with Adipogenesis, Lipid Synthesis, Heat Generation, and Fatty Acid Oxidation in 3T3-L1 Adipocytes

**Figure 3:**
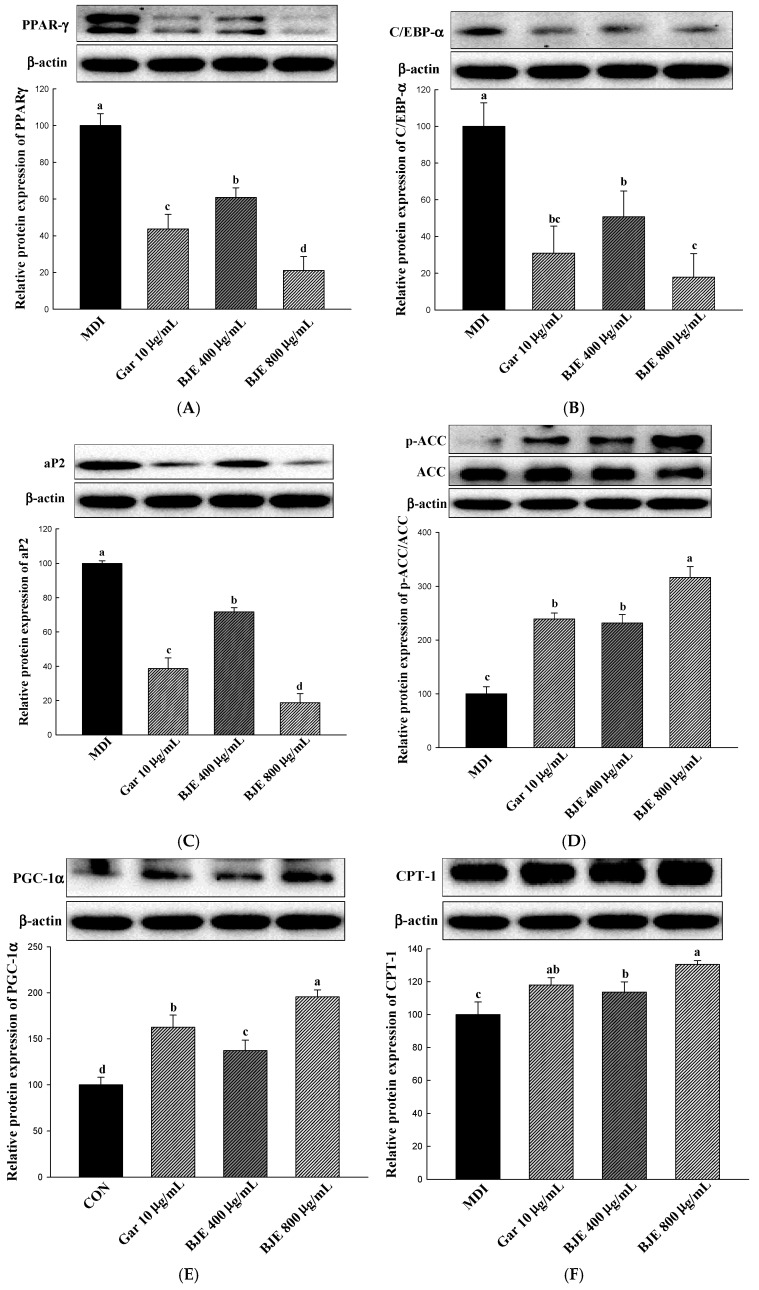
Effect of BJE on the expression of proteins related to adipogenic, lipid synthesis, fatty acid oxidation, and heat generation in 3T3-L1. PPAR-γ (**A**). C/EBP-α (**B**). aP2 (**C**). p-ACC/ACC (**D**). PGC-1α (**E**). CPT-1 (**F**). All results are presented as the mean ± SD of three independent in triplicate. Bars with different letters indicate significant differences at *p* < 0.05 using Duncan’s multiple range test.

This study evaluated alterations in the protein levels of important genes related to the process of adipogenesis, including PPAR-γ, C/EBP-α, and aP2, to clarify the mechanism through which BJE inhibits lipid accumulation (Figure 3A–C). Consequently, within cells exposed to BJE, the levels of C/EBP-α, PPAR-γ, and aP2 expression exhibited a significant decrease with escalating concentrations. To examine the impact of BJE on the protein expression associated with lipid synthesis, the levels of p-ACC/ACC expression were assessed (Figure 3D). In the present study, BJE effectively inhibited lipid synthesis by increasing p-ACC/ACC expression. In order to examine the influence of BJE on the protein expression related to the process of lipolysis, the concentrations of CPT-1 and PGC-1α were evaluated (Figure 3E,F). In the experimental groups treated with BJE, there was a notable increase in the levels of proteins associated with the lipolytic effect with increasing concentrations, in contrast to the MDI group.

### 3.4. Effect of BJE on Body Weight, Food Efficiency, Adipose Tissue Mass, Organ Mass, and Adipose Tissue Size in HFD-Induced Obese C57BL/6J Mice

**Figure 4:**
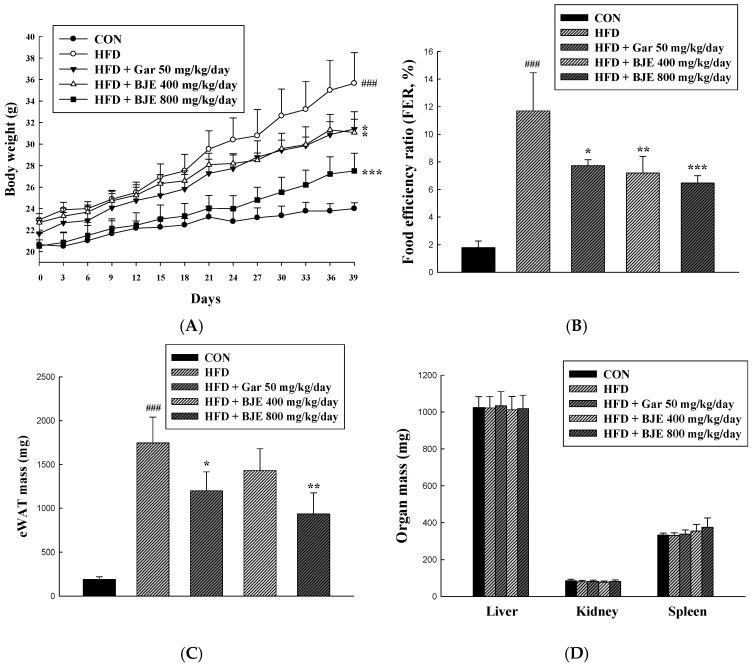

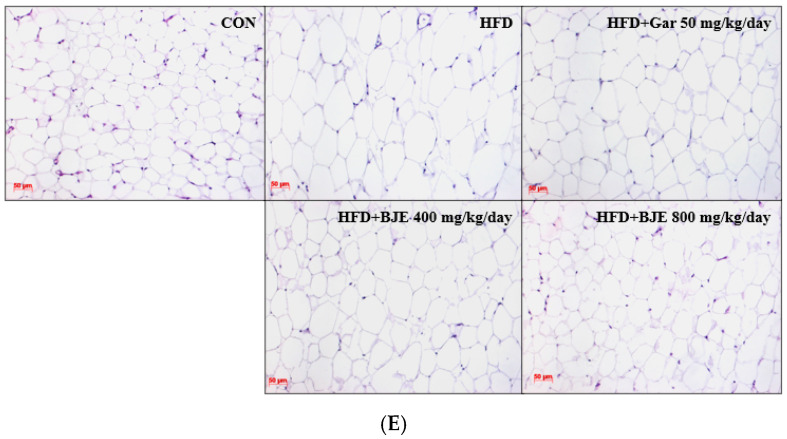
Effect of BJE on body weight, food efficiency, adipose tissue mass, organ mass, and adipose tissue size in HFD-induced obese C57BL/6J mice. eWAT were subjected to staining with H&E and subsequently examined using a microscope. Changes in total body weight (**A**). Food efficiency ratio (**B**). eWAT weight (**C**). Other tissue weight (**D**). Histology of the eWAT (**E**). All values are expressed as the mean ± SD, and statistical analyses were performed using Dunnett’s *t*-test. ### *p* < 0.001 vs. Control group; * *p* < 0.05, ** *p* < 0.01, and *** *p* < 0.001 vs. HFD group.

To examine the potential anti-obesity properties of BJE, mice with diet-induced obesity were treated with either 50 mg/kg/day of Gar (HFD + Gar 50 mg/kg/day), 400 mg/kg/day of BJE (HFD + BJE 400 mg/kg/day), or 800 mg/kg/day of BJE (HFD + 800 mg/kg/day) over a period of 6 weeks. Body weight assessments were conducted weekly over the course of the study period (Figure 4A). As anticipated, the consumption of HFD led to obesity in C57BL/6J mice. The group that consumed HFD showed notable elevations in body weight, FER, and mass of eWAT in comparison to the CON group. However, the body weight, FER, and eWAT mass of the BJE group, who received the oral administration of BJE, exhibited a significant decrease with increasing concentrations of BJE, in contrast to the HFD group. Furthermore, histological analysis revealed a significant increase in the dimensions of adipocytes in the HFD group. It was observed that the alterations in adipocyte dimensions induced by the HFD were effectively reversed by the administration of BJE (Figure 4E). No significant differences in organ mass were detected across the experimental groups (Figure 4D).

### 3.5. Effect of BJE on Serum Biochemical Indexes in HFD-Induced Obese C57BL/6J Mice

Blood samples were obtained from all mice, followed by the separation of serum for the purpose of observing biochemical alterations within the serum (Table 2). To examine the impact of BJE on fatty liver disease, changes in serum concentrations of ALT and AST were assessed. Elevated concentrations of ALT and AST were noted in the HFD group in comparison to the CON group. However, a notable reduction in the concentrations of both ALT and AST was observed in all BJE groups compared to the HFD group. To assess alterations in serum lipid levels resulting from BJE treatment, TC and TG concentrations were evaluated. The HFD group exhibited a significant increase in TC and TG concentrations in comparison to the CON group. Conversely, the BJE group demonstrated a significant reduction in TC and TG concentrations relative to the HFD group with increasing concentrations.

### 3.6. The Effect of BJE on the Regulation of Protein Expression Associated with Adipogenesis, Lipid Synthesis, Heat Generation, and Fatty Acid Oxidation in HFD-Induced Obese Mice

**Figure 5:**
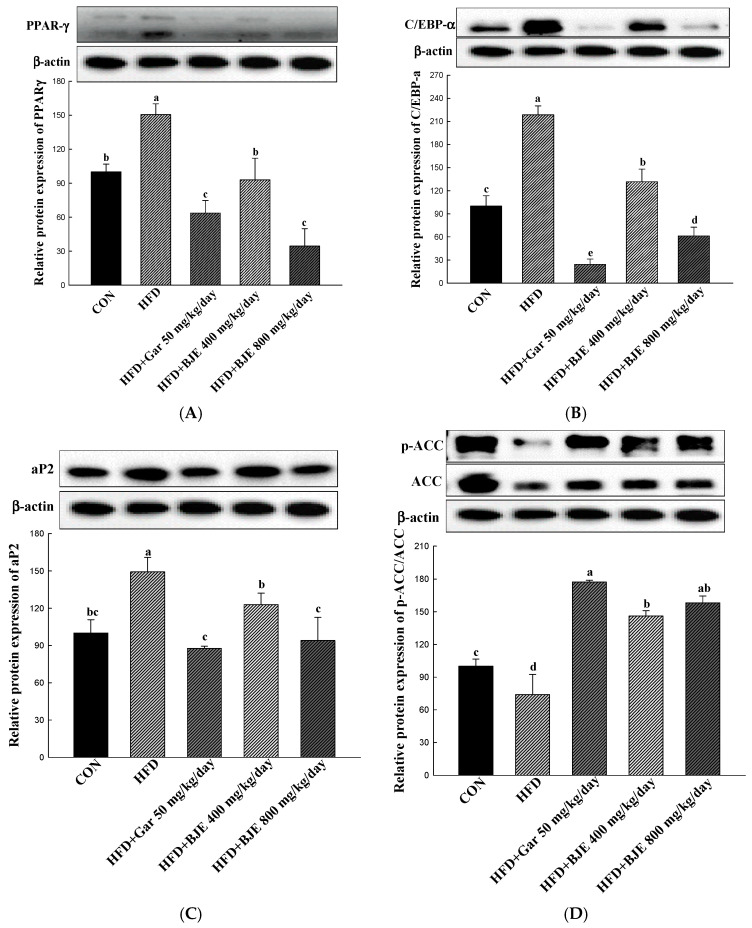

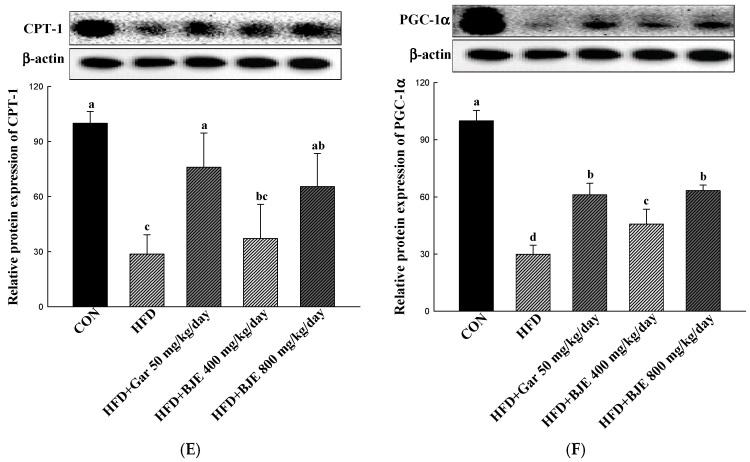
Effect of BJE on the expression of proteins related to adipogenesis, lipid synthesis, fatty acid oxidation, and heat generation in HFD-induced obese C57BL/6J mice. PPAR-γ (**A**). C/EBP-α (**B**). aP2 (**C**). p-ACC/ACC (**D**). PGC-1α (**E**). CPT-1 (**F**). All results are presented as the mean ± SD of 3 independent in triplicate. Bars with different letters indicate significant differences at *p* < 0.05 using Duncan’s multiple range test.

Our study demonstrated that BJE effectively inhibited the process of adipogenesis in differentiated 3T3-L1 adipocytes by reducing the expression levels of PPAR-γ, C/EBP-α, and ap2 (Figure 3A–C). Consistent with these results, the levels of PPAR-γ, C/EBP-α, and ap2 expression were notably decreased in the eWAT obtained from BJE groups compared with those from HFD groups (Figure 5A–C). Furthermore, to assess whether BJE could regulate lipid synthesis-related protein expression in vivo, we measured p-ACC/ACC expression levels in the eWAT. We confirmed that lipid synthesis was suppressed in the BJE group by significantly increasing p-ACC/ACC expression compared to the HFD group (Figure 5D). Finally, the gene expression related to fatty acid oxidation and heat generation was measured. BJE promoted the upregulation of PGC-1α and CPT-1 in vivo, which was decreased in the HFD group (Figure 5E,F). In vivo protein expression results are consistent with in vitro protein expression results.

## 4. Discussion

Several studies have focused on both anti-obesity drugs and functional foods [32,33]. Moreover, many studies have aimed to verify the efficacy of natural substances in functional foods and anti-obesity drugs, with the intention of reducing the various side effects associated with commercially available anti-obesity drugs [34,35,36]. This research was conducted to confirm the in vivo anti-obesity effect in an animal model, expanding on the results of prior in vitro investigations on the inhibition of lipid accumulation by BJE with an optimized sinigrin content [30].

Sinigrin from *Brassica juncea* has been documented to exhibit various beneficial properties such as anticancer, anti-inflammatory, antibacterial, antifungal, and antioxidant effects. In our previous studies, BJE extracted under optimized sinigrin extraction conditions was reported to have anti-obesity effects in vitro [28,29,30]. Therefore, we prepared BJE using an optimized extraction method referring to previous studies and analyzed the content of sinigrin, the main active ingredient of *Brassica juncea*, using HPLC. The sinigrin content of BJE used in this study was 18.0 mg/g.

3T3-L1 preadipocytes were treated with MDI to induce their differentiation into adipocytes. At the same time, the cytotoxicity of BJE in 3T3-L1 adipocytes was confirmed via XTT analysis, but no cytotoxicity was found at all concentrations investigated (100, 200, 400, and 800 μg/mL). The inhibitory effect of BJE (100, 200, 400, and 800 μg/mL) on adipocyte differentiation was investigated. The morphological features of the cells and stained adipocytes were assessed quantitatively using Oil Red O (ORO) staining. This study showed a reduction in both the quantity and dimensions of adipocytes, as well as a decrease in ORO intensity, in correlation with the concentration of BJE, when compared to the MDI group. The in vitro anti-obesity mechanism of BJE was investigated via Western blotting. PPAR-γ, C/EBP-α, and ap2 are crucial factors in the transformation of preadipocytes into fully developed adipocytes [37]. In a previous study, BJE was reported to inhibit lipid accumulation by downregulating protein levels of PPAR-γ, C/EBP-α, and ap2, aligning with the findings of the current study [30]. ACC regulates the condensation of lipids and is integral in the overall regulation of energy metabolism [38]. Phosphorylation leads to the inactivation of this protein, resulting in the inhibition of ACC activity. This process can effectively suppress lipid production and potentially prevent obesity [39]. BJE inhibited ACC activation by promoting ACC phosphorylation. CPT-1 serves as a critical modulator of fatty acid metabolism via its control of malonyl-CoA, which is the initial intermediate in lipid synthesis [40]. PGC-1α is acknowledged as a significant inducer of mitochondrial biogenesis in adipocytes [41]. According to our results, BJE exerts its anti-obesity effects by regulating CPT-1 and PGC-1α to increase fatty acid oxidation and heat generation.

Based on the in vitro results, an animal study was undertaken to evaluate the in vivo efficacy of BJE in combating obesity. HFD is widely used and acknowledged in nutritional studies as an effective method for inducing obesity and fat deposition in animal models [42]. Excessive consumption of HFD can lead to weight gain due to white adipose tissue accumulation [43]. As anticipated, mice that were provided with an HFD exhibited obesity and demonstrated increased eWAT compared to mice that were given a standard control diet. However, orally administering BJE for 6 weeks to HFD-fed mice significantly decreased both adipocyte size in eWAT and body weight. Obesity induced by HFD is correlated with elevated levels of triglycerides and cholesterol in the blood [44]. In this study, BJE decreased serum TC and TG. Western blotting was conducted to examine the in vivo mechanisms underlying the anti-obesity properties of BJE. HFD-induced obesity is associated with high expression of PPAR-γ, C/EBP-α, and ap2 [14]. PPAR-γ, C/EBP-α, and aP2 showed low expression levels Within the adipose tissue of the epididymis in mice administered orally with BJE. ACC was activated in HFD-fed mice and promoted adipogenesis [45]. BJE treatment inhibited ACC activation by restoring ACC phosphorylation in the eWAT of HFD-fed mice. However, in HFD-fed mice, the expression of PGC-1α, which is involved in heat generation, and CPT-1, a key enzyme responsible for facilitating the oxidation of fatty acids [46,47]. In this research endeavor, we investigated the impact of BJE treatment on the expression of proteins related to heat generation and fatty acid oxidation in vivo. Here, we showed that BJE administration induced PGC-1α and CPT-1 expression in HFD-fed obese mice. Therefore, BJE treatment may inhibit lipid accumulation by upregulating the heat generation and expression of fatty acid oxidation-related proteins.

Overall, these results confirmed that BJE prevented obesity induced by HFD in C57BL/6J mice. Western blot analysis demonstrated that the anti-obesity properties of BJE were linked to the suppression of adipogenesis and lipid synthesis, along with the stimulation of fatty acid oxidation and heat generation. Therefore, BJE may be a promising source of functional materials that could be utilized in the prevention and management of obesity.

## 5. Conclusions

The research demonstrates that BJE can inhibit lipid accumulation in 3T3-L1 and decrease body weight and eWAT mass in obese mice fed an HFD. These outcomes are accomplished by inhibiting adipogenic proteins (C/EBP-α, PPAR-γ, and aP2), activating the phosphorylation of the lipid synthesis protein (ACC), and increasing the levels of fatty acid oxidation protein (CPT-1) and thermogenesis-related protein (PGC-1α). Therefore, our data suggest that BJE administration improves HFD-induced obesity by suppressing the formation of adipocytes and promoting the processes of heat generation and fatty acid oxidation. However, it is imperative to conduct suitable human trials to investigate its use as an alternative to anti-obesity drugs for the purpose of controlling obesity.

## Figures and Tables

**Table 1:** HPLC condition for sinigrin analysis.

Instrument	Conditions
Column	CAPCELL PAK C_18_, UG120 (5.0 μm, 4.6 mm × 250 mm)
Column temp.	30 °C
Mobile phase (isocratic)	Isocratic HPLC water containing 0.1 M ammonium sulfate
Detector	PDA detector (228 nm)
Flow rate	1.0 mL/min
Injection volume	40 μL
Run time	30 min

**Table 2:** Effects of BJE on serum biochemistry in HFD-induced obese C57BL/6J mice.

Parameters	Groups				
	CON	HFD	HFD + Gar 50 mg/kg/day	HFD + BJE 400 mg/kg/day	HFD + BJE 800 mg/kg/day
ALT (U/L)	32.00 ± 5.24	46.00 ± 12.36	38.00 ± 8.02	30.00 ± 5.82 *	29.00 ± 1.23 *
AST (U/L)	117.00 ± 20.37	243.00 ± 74.80 ^#^	134.00 ± 64.60	126.00 ± 58.65 *	110.00 ± 27.00 *
TC (mg/dL)	106.00 ± 4.53	161.00 ± 9.66 ^###^	173.00 ± 11.06	163.00 ± 12.16	142.00 ± 5.64 *
TG (mg/dL)	40.00 ± 7.65	86.00 ± 5.81 ^###^	64.00 ± 8.71 **	71.00 ± 9.93	57.00 ± 7.86 ***

All values are expressed as the mean ± SD, and statistical analyses were performed using Dunnett’s *t*-test. # *p* < 0.05, ### *p* < 0.001 vs. Control group; * *p* < 0.05, ** *p* < 0.01, and *** *p* < 0.001 vs. HFD group. ALT: alanine aminotransferase, AST: aspartate aminotransferase, TC: total cholesterol, TG: total triglyceride.

## Data Availability

The data presented in this study are available within this article.
